# New Targeted Molecular Therapies for Dedifferentiated Thyroid Cancer

**DOI:** 10.1155/2010/921682

**Published:** 2010-06-06

**Authors:** Alessandro Antonelli, Clodoveo Ferri, Silvia Martina Ferrari, Marco Sebastiani, Michele Colaci, Ilaria Ruffilli, Poupak Fallahi

**Affiliations:** ^1^Department of Internal Medicine, Metabolism Unit, University of Pisa School of Medicine, Via Roma, 67, 56100 Pisa, Italy; ^2^Rheumatology Unit, Department of Internal Medicine, School of Medicine, University of Modena and Reggio Emilia, Via del Pozzo, 71, 41100 Modena, Italy

## Abstract

Dedifferentiated thyroid cancer (DeTC) derived from follicular epithelium is often incurable because it does not respond to radioiodine, radiotherapy, or chemotherapy. In cases, RET/PTC rearrangements are found in 30%–40%, RAS mutations in about 10%, and BRAF mutations in around 40%–50%, with no overlap between these mutations results in papillary thyroid cancer, while a higher prevalence of BRAF mutations (up to 70%) has been observed in DeTC. The identification of these activating mutations in DeTC makes this malignancy an excellent model to examine the effect of tyrosine kinase inhibitors (TKIs). Clinical trials with several TKIs targeting RET, and to a lesser extent BRAF, and other TKRs have shown positive results, with about one-third of DeTC showing a reduction in tumor size up to 50%, with the longest treatment duration of approximately three-four years. Angiogenesis inhibitors have also shown promising activity in DeTC. Progress is being made toward effective targeted DeTC therapy. The possibility of testing the sensitivity of primary DeTC cells from each subject to different TKIs could increase the effectiveness of the treatment.

## 1. Introduction

Thyroid carcinoma is the most prevalent endocrine malignancy and accounts for 1% of all human cancers. Approximately 90% of thyroid malignancies are well-differentiated thyroid carcinomas, which are classified as papillary or follicular based on histopathological criteria. Even though differentiated thyroid carcinomas are usually curable by the combination of surgery, radioiodine ablation, and thyroid-stimulating hormone suppressive therapy, recurrence occurs in 20%–40% of patients [1, 2]. During tumor progression, cellular dedifferentiation occurs in up to 5% of cases and is usually accompanied by more aggressive growth, metastatic spread, and loss of iodide uptake ability, making the tumor resistant to the traditional therapeutic modalities and radioiodine. Conventional chemotherapy and radiotherapy have a modest, if any, effect on advanced dedifferentiated thyroid cancer (DeTC) [3], which is responsible for a large number of deaths attributed to thyroid cancer. Therefore, advanced DeTC represents a therapeutic dilemma and is considered a critical area of research.

## 2. Molecular Changes in DeTC

Iodide trapping is a thyrotropin- (TSH-) regulated mechanism involving an energy-dependent transport mediated by the Sodium/Iodine symporter (NIS) [3, 4] at the basolateral surface of the thyrocyte and passive transport at the apical surface, where a role has been suggested for the Pendred syndrome (PDS) gene. At the apical surface the iodide is organified by thyroperoxidase (TPO) and conjugated to tyrosine residues on thyroglobulin (Tg). A major drop in NIS transcripts has been demonstrated in primary and metastatic thyroid tumors by comparison with normal tissues, but this is far less evident in metastases with no radioiodine (131I) uptake than in primary cancers and metastases able to trap 131I, suggesting that mechanisms other than a mere genetic control over NIS transcription might be involved in this failure to trap 131I [5]. Tg, TPO, and PDS gene expressions are lower in thyroid cancers than in normal tissues. A significant gene expression decrease of such molecules was also found in metastases with no 131I uptake by comparison with either primary cancers or metastases with a positive 131I whole-body scan (WBS). These differences could mean that a demonstrable 131I uptake by thyroid cancers requires not only a functional and correctly located NIS but also the full machinery responsible for iodide retention in the cell. Indirect confirmation of this hypothesis seems to come from gene therapy studies, where the NIS gene was introduced in nonthyroid cancer cells to promote 131I uptake and induce cytotoxicity. Such reports demonstrated that although NIS delivery in the target cells was followed by an efficient iodine uptake, therapeutic effects were only observed when high doses of radioiodine (beyond the ranges used in humans) were administered [5]. For cancers failing to trap 131I, the availability of imaging procedures to detect metastatic disease is crucial to the use of surgery with a curative intent [1]. Several reports have demonstrated the effectiveness of fludeoxyglucose-positron emission tomography (FDG-PET) in the postoperative management of thyroid cancers, particularly in patients with high serum Tg levels and negative 131I WBS. Such effectiveness is consistent with different molecular studies showing that the higher glucose consumption in primary cancers is accompanied by an increase in its transmembrane transport due to GLUT-1 overexpression; this increase correlates with more aggressive histotypes and the presence of local and distant metastases. The FDG-PET scan's sensitivity might be improved by TSH stimulation. Preliminary in vitro studies have demonstrated that TSH stimulation in FRTL-5 cells is followed by an increased glucose uptake, and subsequent in vivo studies have demonstrated that the FDG-PET scan became more accurate after administering recombinant human TSH, revealing lesions not seen in conditions of TSH suppression and inducing changes in the extent of surgery and ameliorating management and outcome [1].

Moreover, recently it has been shown that BRAF mutation in papillary thyroid cancer is associated with a more aggressive phenotype and less differentiated state due to decreased expression of iodide-metabolizing [6] and sodium iodide symporter genes [7].

Furthermore, the BRAF V600E oncogene induces transforming growth factor-beta secretion leading to sodium iodide symporter repression and increased malignancy in thyroid cancer [8], and targeted expression of BRAF V600E in thyroid cells of transgenic mice results in papillary thyroid cancers that undergo dedifferentiation [9].

## 3. Oncogenes

Molecular abnormalities, believed to cause thyroid cancer, have been recorded in papillary and follicular thyroid carcinomas. In 80% of papillary thyroid carcinomas (PTCs), mutations have been noted in genes that encode signaling molecules of the mitogen-activated protein kinase (MAPK) pathway [10]. In adult sporadic papillary carcinomas, RET/PTC rearrangements are found in 30%–40% of cases, RAS mutations in about 10% of cases, and BRAF mutations in around 40% of cases, with no overlap between these mutations [5, 11]. In radiation-induced papillary carcinomas and those in childhood, RET/PTC rearrangements have been seen in 50%–80% of cases and BRAF mutations in 10% of cases [5, 11]. Inhibitors of BRAF kinase block the growth of thyroid cancer cells that have RET/PTC or BRAF mutations [12]. 

In a recent study, MEK inhibitor CI-1040 has been found to abrogate tumor growth in BRAF mutant xenografts derived from various tumor types [13].

Furthermore, a recent study demonstrated that treatment of cells with MEK inhibitors could restore the expression of Tg and NIS [14]. 

In addition to the activation of the MAPK pathway, these mutations and most tyrosine kinase receptors can also result in the activation of the phosphatidylinositol 3-kinase [15, 16].

Of the several forms of RET/PTC, RET/PTC1 and RET/PTC3 are the most frequently found in PTC [17]. RET/PTC tyrosine kinases are constitutively active and trigger the activation of signaling pathways by interacting with and phosphorylating several signaling molecules.

Activation of RET/PTC in cultured thyroid cells results in downregulation of expression of Tg and NIS and cell dedifferentiation [18, 19]. These effects of RET/PTC activation require signaling along the MAPK pathway [20] and, more specifically, the presence of the functional BRAF kinase [21]. Indeed, BRAF silencing in cultured thyroid cells reverses the RET/PTC-induced effects such as ERK phosphorylation [18]. However, signaling from the wild-type RET receptor and its truncated RET/PTC forms is also known to activate a number of other pathways, particularly the phosphatidylinositol-3 kinase/AKT pathway, which may also contribute to its biological effects [22, 23].

BRAF mutations have been associated with more aggressive and less differentiated papillary tumors, and this is consistent with the inhibition of thyroid-tumor cell growth induced by the blockade of BRAF kinase. 

Recently, the effect of a BRAF small inhibitory RNA construct and the BRAF kinase inhibitor AAL881 on both BRAF wild-type and mutant thyroid carcinoma cell lines was evaluated. A small inhibitory RNA construct targeting the expression of both wild-type BRAF and BRAF V600E induced a comparable reduction of viability in both wild-type and BRAF V600E mutant cancer cells. Interestingly, AAL881 inhibited MEK and ERK phosphorylation and induced apoptosis preferentially in BRAF V600E-harboring cells than wild-type ones, possibly because of better inhibitory activity against BRAF V600E [24].

Recently, it was investigated whether sensitivity to MEK inhibition was determined by oncogene status in 13 human thyroid cancer cell lines: four with BRAF mutations, four RAS, one RET/PTC1, and four wild type. Thyroid cancers with BRAF mutation were preferentially sensitive to MEK inhibitors (PD0325901, AZD6244) whereas tumors with other MEK-ERK effector pathway gene mutations have variable responses [25].

Overexpression of tyrosine kinase receptors has been noted in thyroid cancer cells, including those for fibroblast growth factor, epidermal growth factor (EGF), hepatocyte growth factor (c-Met), vascular endothelial growth factor (VEGF), insulin, and insulin growth factor 1. The ligands for these receptors are also often overexpressed. Angiogenesis might also have a crucial role in the development of these hypervascularised tumors, and the expression of angiogenic markers can have prognostic significance [26–28]. In experimental models, antivascular treatment blocks the growth of DeTC [29, 30].

## 4. New Molecular Targeted Therapies in Advanced Thyroid Cancer

Activated oncogenes are highly attractive targets for the development of new specific anticancer agents. A growing number of drugs that target an activated oncogene are already showing benefits to patients in clinical applications, thereby demonstrating the potential of oncogene inhibition in human cancer. In addition, other drugs that target activated or overexpressed oncogenes at different mechanistic levels have recently been approved, such as the EGF receptor (EGFR) inhibitors and VEGF and VEGF receptor-2 (VEGFR-2) inhibitors, which can prevent tumor angiogenesis [31, 32]. All of these examples illustrate that patients with cancer can significantly profit from the development of targeted therapies, especially when an activated oncogene is proven to be the underlying cause for a given malignancy. However, all these new small molecules demonstrated a higher activity when administered in combination with chemotherapeutic drugs [32]. 

One highly promising target for the development of a selective therapy for the treatment of patients with PTC is the activated RET oncogene [33]. The RET/PTC family of chimeric oncogenes result from several rearrangements involving the RET gene of chromosome 10q; they are specific to papillary carcinomas and are present in up to 77% of these tumors. The proteins product of the RET/PTC oncogenes all contain the intracellular tyrosine kinase domain of the normal RET proto-oncogene product and can therefore be immunohistochemically detected with an antibody to the carboxy terminus of RET [34], even if this technique may be problematic. The search for new therapeutic options, therefore, becomes increasingly important, especially for patients with metastatic disease. Since somatic mutations in RET are known to be causative for the development of PTC, activated RET is the most obvious target for a new strategy to treat this disease. 

Moreover, there are recent experimental evidences of autocrine activation of EGFRs and VEGFRs in PTC cell lines (e.g., TPC1 cells) [35] that suggest a particular rationale for the use of tyrosine kinase inhibitors with dual modes of action such as ZD6474 [36] and SU11248 [33]. Furthermore, these compounds can also effectively act through the inhibition of tumor angiogenesis, a key process of the neoplastic growth in thyroid cancers [37].

To date a number of structurally different compounds have been reported as ATP-competitive inhibitors of the tyrosine kinase activity of RET. They include quinazolines, such as ZD6474 [36], indolin-2-ones, that are RPI-1 [38] and SU5416 [39], and pyrazolo[3,4-d]pyrimidines, like PP1 [40] and PP2 [41], all showing various degrees of efficacy and specificity with respect to other tyrosine kinases. The pyrazolopyrimidine derivatives PP1 and PP2, in particular, proved to be the most potent compounds, showing IC50 values of 80 and 100 nM, respectively. Moreover, they are also good inhibitors of protooncogene Src, a key downstream RET effector. Therefore, these compounds are proposed for a multiple-signal transduction strategy, which may have therapeutic potential for the management of thyroid carcinomas. The anilinoquinazoline ZD6474 is an oral small-molecule inhibitor of VEGFR and EGFR tyrosine kinases and is currently implicated in several phase I and phase II trials [42]. ZD6474 was also found to target the RET kinase in mammalian cell cultures and to efficiently block the invasive growth of cancer cells [36]. However, these new small molecules are highly effective in combination with standard chemotherapy. Among chemotherapeutic drugs, preclinical studies using irinotecan seem to be very promising in different types of thyroid carcinomas such as anaplastic and medullary ones, especially in combination with treatments with anti EGFR antibodies (cetuximab) and small molecules inhibiting tyrosine kinases (PTK787) [43–45].

## 5. Clinical Activity of New Molecular Targeted Therapies in Advanced Thyroid Cancer

The clinical activity of several compounds, including motesanib diphosphate (AMG 706), BAY 43-9006, ZD 64-74, and AG-013736, in DeTC is being studied in phase II trials [33, 46–49], with preliminary studies recording partial response and stabilization [50–52]. These molecules inhibit several targets, including RET tyrosine kinase, VEGFR1, VEGFR2, and VEGFR3, and have an antiangiogenic effect. BAY 43-9006 also inhibits BRAF kinase. Other targets include membrane receptor kinases, such as those for EGF, platelet-derived growth factor, and c-Met. Any clinical effect of each of these drugs could be related to the inhibition of the MAPK pathway and of angiogenesis and possibly to the inhibition of other targets.

The results obtained with some of these agents are interesting. Axitinib, a selective inhibitor of VEGFR 1, 2, and 3, was investigated in the therapy of sixty patients with advanced thyroid cancer. Partial responses were observed in 18 patients. Stable disease lasting > or = 16 weeks was reported in another 23 patients. The authors conclude that Axitinib has antitumor activity in all histologic subtypes of advanced thyroid cancer [53].

 AMG 706 (a novel oral inhibitor of VEGFR, platelet-derived growth-factor receptor, and KIT) was used in the treatment (125 mg administered orally once daily) of 93 patients with progressive, locally advanced or metastatic, radioiodine-resistant differentiated thyroid cancer. The objective response rate was 14%; stable disease was achieved in 67% of the patients, and stable disease was maintained for 24 weeks or longer in 35%. The authors concluded that AMG 706 can induce partial responses in patients with advanced or metastatic differentiated thyroid cancer [54].

An open-label phase II trial with sorafenib (that inhibits a spectrum of kinases including Raf kinase, VEGFR, platelet-derived growth factor receptor, and RET tyrosine kinases) was conducted in patients with advanced, metastatic, iodine-refractory thyroid carcinoma. Thirty patients were treated for a minimum of 16 weeks. Seven patients (23%) had a partial response lasting 18 to 84 weeks. Sixteen patients (53%) had stable disease lasting 14 to 89 weeks. The authors concluded that Sorafenib has clinically relevant antitumor activity in patients with metastatic, iodine-refractory thyroid carcinoma, with an overall clinical benefit rate (partial response + stable disease) of 77% [55]. 

In another study of 41 PTC patients treated with Sorafenib, six patients had a partial response (PR 15%; 95% CI, 6 to 29) and 23 patients (56%; 95% CI, 40 to 72) had stable disease longer than 6 months. The authors concluded that Sorafenib is reasonably well-tolerated therapy with clinical and biologic antitumor activity in metastatic PTC [56].

## 6. Choice of the Optimal Treatment

New therapeutic approaches against DeTC are under development. However, more research is needed to finally identify therapies able to control and to cure this disease. 

There are still some limitations in the selective use of novel compounds. There might be several potential targets in a given tumor, and most compounds can hit multiple targets, so the most important target for a given tumor might remain uncertain. Even when potential targets (such as RET) are present in the tumor tissue, tumor response might be observed in only a fraction of patients. A lack of response can occur, for instance, because target inhibition raises the activity of compensatory signal pathways, which, in turn, rescues tumor cell growth. Moreover, the possibility of testing the sensitivity of primary DeTC cells from each subject to different tyrosine kinase inhibitors could increase the effectiveness of the treatment; in fact, in vitro chemosensitivity tests are able to predict in vivo effectiveness in 60% of cases [57]. While, it is well known that a negative chemosensitivity test in vitro is associated with a 90% of ineffectiveness of the treatment in vivo [57], allowing the administration of inactive chemotherapeutics to these patients to be avoided [58–60].
